# In Situ Formation of HAc/Ac^−^ Buffer Pair via Sodium Acetate Addition Enhances Two-Stage Continuous Biohythane Production from Xylose: Process Performance and Microbial Community Dynamics

**DOI:** 10.3390/microorganisms14102389

**Published:** 2026-10-19

**Authors:** Xiaoyi Feng, Kaini Dong, Qiulan Xie, Lili Dong, Jiwen Wu, Guangli Cao

**Affiliations:** 1 State Key Laboratory of Urban Water Resources and Environment, School of Environment, Harbin Institute of Technology, Harbin 150090, China; 2 Key Laboratory of Agro-Forestry Environmental Processes and Ecological Regulation, School of Environmental Science and Engineering, Hainan University, Haikou 570228, China

**Keywords:** two-stage biohythane production, in situ HAc/NaAc buffer system, xylose wastewater, process stability, microbial community succession

## Abstract

Biohythane has attracted growing interest as an eco-friendly biofuel. However, its production stability is often compromised by rapid acidification during the dark fermentation hydrogen production stage. Acidification intensifies proton competition between ATP synthesis and H_2_ evolution, leading to electron transfer inefficiency and metabolic disruption. While buffering systems can stabilize pH, the mechanisms by which adaptive buffers regulate microbial metabolism in two-stage biohythane fermentation remain unclear. In this study, sodium acetate (NaAc) was supplemented to a xylose-fed two-stage fermentation process, where it formed an in situ HAc/Ac^-^ buffer pair with the acetic acid generated endogenously during fermentation, achieving efficient continuous biohythane (H_2_ + CH_4_) production. The optimized semi-continuous system attained complete xylose degradation, with a maximum biohythane yield of 10.99 mol/mol, comprising 21.1% H_2_ and 78.9% CH_4_, representing an approximately 60% increase over the control. Volatile fatty acid and pH profiles indicated that NaAc addition provided a critical physicochemical environment that ensured high operational efficiency. Microbial analysis revealed that *Caproiciproducens* (52.08%) and *Alcaligenes* (16.94%) dominated the H_2_ production stage, while *Methanoculleus thermophilus* (46.66%) and *Methanothermobacter thermautotrophicus* (37.94%) prevailed in the CH_4_ production stage. This study proposes a feasible process control strategy for the two-stage biohythane fermentation process, which not only mitigates the acidification challenge in biological H_2_ production but also enhances substrate-to-bioenergy conversion efficiency.

## 1. Introduction

Bioenergy has increasingly played a critical role in sustainable transportation as a renewable and eco-friendly substitute for traditional fossil fuels [1]. Hydrogen (H_2_) and methane (CH_4_) represent two of the most prominent energy carriers in modern renewable energy systems, owing to their high energy densities of 142 MJ/kg and 55.5 MJ/kg, respectively [2]. Their mixture, defined as hythane, typically contains 10–25% H_2_ and synergistically combines the merits of both gases, offering superior combustion efficiency and reduced carbon emissions compared to pure methane [3]. From the perspective of waste recycling and renewable energy production pathways, biohythane represents a crucial transitional solution, bridging the gap between traditional methane-based natural gas and the future hydrogen economy [4]. To realize this potential, designing and demonstrating an uninterrupted biohythane production process becomes the most important.

Two-stage anaerobic digestion (TSAD) produces hythane from food waste, organic components of municipal solid waste, agricultural waste, wastewater, etc., offering an eco-friendly substitute for fossil fuels, reducing emissions, and aiding in achieving circular economy goals [5,6]. Compared with traditional single-stage anaerobic digestion systems, the TSAD configuration separates the acidogenic and methanogenic phases into dedicated reactors; thus the first reactor optimizes pH conditions for hydrolytic/acidogenic bacteria to perform hydrolysis, acidification, and acetogenesis, while the second reactor maintains ideal pH ranges specifically for methanogenic archaea to facilitate CH_4_ production [7,8,9]. This method can effectively mitigate inhibition between acidogens and methanogens, preventing reactor failure from acid accumulation while reducing system HRT and sustaining high biogas methane content. Previous studies have shown that maintaining stable pH conditions during the acidification stage is crucial for optimizing process performance and ensuring operational stability in anaerobic digestion systems [10]. In a typical two-stage biohythane production process, since the acidification stage consists of a fast reaction process and short hydraulic retention time, the pH is adjusted by adding an alkali agent, allowing the reactor to be incubated for 1–3 days at pH 4.5–6.5 in the first stage and 10–15 days at pH 6.5–7.8 in the second stage [11,12,13]. Therefore, pH control strategies significantly influence biogas production performance, but their effectiveness varies depending on the specific approach employed. For instance, for the reported maximum gas production yields of 7.98 mL H_2_/g-VS and 204.61 mL CH_4_/g-VS from the two-stage biohythane fermentation of straw residues using HCl or NaOH for pH adjustment between 5.5 and 6.5 [14]. Yellezuome et al. (2024), 2 M NaOH was adopted to regulate a two-stage continuous food waste and chicken manure fermentation system, achieving a peak biohythane yield of 690 mL H_2_/L·d and 690 mL CH_4_/L·d at an OLR of 8 g VS/L·d [15]. Although TSAD has been established for several decades, it still needs to be optimized in light of process stability, inhibition problems, and higher H_2_ yields.

Typically, overall process efficiency is predominantly governed by first-stage fermentation performance. Theoretically, the complete bioconversion of glucose could yield 12 mol H_2_/mol glucose, yet practical yields do not exceed 4 mol H_2_/mol glucose due to multiple limiting factors [16,17]. The reported peak hydrogen production level remains 3.47 mol H_2_/mol glucose, constrained by process instability, reactor design, acid accumulation, competitive consortia, substrate composition, and nutrient availability [16,17]. A well-documented inhibition phenomenon arises from the rapid pH decline caused by the accumulation and dissociation of acidic metabolites (e.g., acetate, butyrate), leading to metabolic suppression and hydrogenase inhibition, especially at pH below 5.0, resulting in a detrimental shift toward non-hydrogenic metabolites [18,19]. To mitigate acidification, alkaline or buffering agents such as NaHCO_3_ have been used, with studies showing optimal H_2_ productivity at specific concentrations (e.g., 3.0 g/L NaHCO_3_) [20]. However, strong alkalis like NaOH or KOH can disrupt biochemical equilibrium and reduce buffer capacity [21,22]. Thus, innovative buffering strategies tailored to substrate characteristics and dynamic process parameters are needed. Our previous work demonstrated that sodium acetate (NaAc) significantly enhanced H_2_ production in cellulose-based systems, being 2.84 times higher than the control and 57.7% higher than that for NaOH-treated groups, offering a novel pH regulation approach [18]. Nevertheless, the long-term stability of NaAc-mediated pH control and its interactions with substrates in continuous biohythane systems remain unverified, representing critical barriers to scale-up.

In this study, both the semi-continuous operation of a two-stage biohythane fermentation system using xylose as the substrate and the repeated batch experiment were carried out to (i) demonstrate the stability of long-term semi-continuous H_2_ and CH_4_ production from xylose by added NaAc to regulate pH; (ii) clarify the effect of the HAc/NaAc buffer system on the pH and distribution of metabolites, especially the dissociation characteristics of VFAs; and (iii) elucidate the succession characteristics of H_2_-producing and CH_4_-producing microbial community dynamics, especially explaining how community shifts are triggered by backflow from the methanogenic to H_2_-producing stage.

## 2. Materials and Methods

### 2.1. Inoculum and Substrate

The inoculum used in the H_2_ production stage is a domesticated mixed bacterial culture obtained from previous experiments that can degrade xylose and produce H_2_ under thermophilic conditions. At the generic level, the dominant strain of this mixed bacterium was *DSM—571* (99.94%) of *Thermoanaerobacium* [23]. The inoculum used in the CH_4_ production stage is biogas slurry from previous experiments that can be degraded and discharged during the H_2_ production stage and produce CH_4_ under thermophilic conditions [24]. Xylose (Shanghai Yuanye Bio-Technology Co., Ltd., Shanghai, China) was used as a carbon source. The bacterial culture was inoculated into modified ATCC 1191 medium (MA), and the specific characteristics of MA and enrichment methods are detailed in Text S1.

### 2.2. Batch Test of Fermentation H_2_ Production

Tests with different xylose concentrations (2.5, 5, 10, 15, and 20 g/L) for biohydrogen production were all carried out in 100 mL anaerobic bottles with a 50 mL working volume; the specific components of the medium are shown in Text S1, and the initial pH of the medium was adjusted to 7.15 by 5 M NaOH. Then, different concentrations of NaAc (0.1, 0.15, 0.2, and 0.25 mol/L) were added into the media, and then high-purity nitrogen gas (99.999%) was purged into sealed vials with needles inserted through stoppers for 5 min to provide anaerobic conditions. Then, anaerobic bottles were placed in a high-pressure steam sterilization pot at 115 °C for 30 min. Subsequently, the inocula obtained in the phase of exponential growth were inoculated into every bottle at a concentration of 2% (*v*/*v*). The bottles were incubated at 60°C in a constant-temperature water bath shaker, and the speed was 170 rpm. Gas samples were taken every 24 h by transferring the gas from the bottle headspace to the gas sampling bag, and liquid samples were taken at the end of fermentation. Fermentations in each condition were repeated at least three times.

### 2.3. Semi-Continuous Biohythane Production from Xylose with Buffer Regulation in an Integrated Reactor

Semi-continuous biohythane production was conducted in a novel integrated anaerobic baffled reactor (IABR; total volume: 5.84 L, working volume: 5.4 L, including a 1 L hydrogen-producing area (HPA) and a 4.84 L methane-producing area (MPA)) under the optimized concentrations of xylose and sodium acetate (NaAc), as detailed in Supplementary Table S1 [24]. Prior to startup, the reactor was inoculated with 100% (*v*/*v*) pretreated anaerobic sludge. The process was initiated with a 4-day batch operation of the HPA to enable microbial acclimation and biomass growth. The transition to semi-continuous mode was triggered upon achieving stable H_2_ production, which indicated a consistent gas output with minimal fluctuation. The subsequent operational shifts, including OLR and HRT adjustments, were guided by the gas production profile from the preceding phase, maintaining stability under steady performance or modifying conditions in response to significant variations. The operational parameters are detailed in Supplementary Table S1. The effluent from the HPA served as the substrate for the MPA. During the CH_4_ production phase, the organic loading rate (OLR) was progressively increased, and the hydraulic retention time (HRT) was adjusted accordingly, until a steady operational regime was attained and maintained until the end of the experiment. Briefly, the influent flow rate ranged from 100 to 900 mL/d, corresponding to an HRT of 5–45 d (4.5 L working volume) and a xylose OLR of 0.04–0.70 g/L/d, and stabilized at 600 mL/d (HRT 7.5 d; OLR 0.33 g/L/d) after day 70. No reflux was applied. pH was controlled solely by NaAc supplementation through the in situ HAc/Ac^-^ buffer. Throughout the operation, daily gas production was measured by collecting headspace gas into sampling bags every 24 h. Concurrently, liquid samples were taken from both the HPA and MPA to monitor key performance indicators, including pH, volatile fatty acids (VFAs), and residual xylose concentration.

### 2.4. Analytical Methods

The gases in the H_2_ production stage and the CH_4_ production stage were regularly recorded by using a gas collection bag every 24 h and analyzed using a gas chromatograph (GC9790PLUS, FuLi Inc., Wenling, China) equipped with a thermal conductivity detector [25]. The metabolic products (VFAs and ethanol) were also measured by gas chromatography (GC9790PLUS, FULI Inc., Wenling, China). The pH was detected by HACHHQ40d multi (HACH, Loveland, United States). Xylose in the liquid phase was determined by using a colorimetric phenol–sulphuric acid method [26]. All data were obtained from a single semi-continuous two-stage biohythane production reactor. Because parallel independent biological replicates were not used, error bars and statistical tests are not presented.

The energy conversion efficiency of H_2_ and CH_4_ from xylose was calculated by Equation (1):

(1) $$E=\frac{V_{H_{2}\;\mathrm{or}\;CH_{4}}\times Q_{H_{2}\;\mathrm{or}\;CH_{4}}}{Q_{\mathrm{xylose}}\times m+m_{\mathrm{NaAc}}\times Q_{\mathrm{NaAc}}}\times 100\%$$

where *E* is energy conversion efficiency (%); *V_H_*_2 or *CH*4_ is cumulative H_2_ or CH_4_ production (mL); *Q_H_*_2 or *CH*4_, *Q_xylose_*, and *Q_NaAc_* are the heating values of H_2_ (12.86 J/mL) or CH_4_ (35.9 J/mL), xylose (4000.0 J/g), and NaAc (10,608 J/g) respectively; and m is the amount of xylose and NaAc consumed (g).

The theoretical pH values were calculated based on the measured VFA concentrations (acetate, propionate, butyrate, and valerate) in the fermentation broth. The term “theoretical pH” refers to the pH calculated by solving the charge balance equation under the assumption that proton concentration is governed solely by the dissociation equilibria of these VFAs, reflecting the pH dominated by VFA dissociation in the absence of interference from other buffering systems (e.g., phosphate, ammonium, carbonate). The calculation was performed as follows.

The degree of dissociation for each acid was calculated using the Henderson–Hasselbalch equation shown in Equation (2):

(2) $$\mathrm{pH}=\mathrm{pKa}+\log\frac{\left[A^{-}\right]}{\mathrm{HA}}$$

Mass balance for each component: *C_i_* = [*HA*]*_i_* + [*A*^−^]*_i_*.

Charge balance equation (activity coefficients approximated as unity):

(3) $$\sum i{\left[A^{-}\right]}_{I}+\left[OH^{-}\right]=\left[H^{+}\right]$$

where *C_i_* is the measured total concentration of the *i*-th VFA, and [*HA*]*_i_* and [*A*^−^]*_i_* are the concentrations of its undissociated and dissociated forms, respectively. [*OH*^−^] = *K_w_*/[*H*^+^], with *K_w_* =1.0 × 10^−14^ at 25 °C. The *pKa* values used were 4.76 for acetate, 4.87 for propionate, 4.82 for butyrate, and 4.86 for valerate. The theoretical pH was then determined as −log[*H*^+^] after solving the above equations for [*H*^+^].

### 2.5. Microbial Community Analysis

High-throughput sequencing was conducted to study the abundance and spatial distribution of bacteria in different operating stages of the system on day 22, day 53, and day 73 in the H_2_ production stage and day 46, day 54, and day 73 in the CH_4_ production stage. Subsequently, 5–10 mL samples were taken from the sampling port of the reaction system and placed in centrifuge tubes for sealed storage. The sample volume was controlled at around 3–10 mL. The samples were immediately frozen after being taken out and then stored in a −80 °C refrigerator for subsequent DNA extraction and analysis.

The sequencing of 16S rRNA genes was conducted using Majorbio Bio-Pharm Technology Co.’s Illumina Miseq 2 × 300 bp sequencing system. (Location: Shanghai, China). The forward primer for bacteria was 338F “5′-ACTCCTACGGGAGGCAGCAG-3′”, the reverse primer was 806R “5′-GGACTACHVGGGTWTCTAAT-3’”, the forward primer for archaea was 524F”5′-TGYCAGCCGCCGCGGTAA-3′”, and the reverse primer was 985R”5′-YCCGGCGTTGAVTCCAATT-3′”. The SILVA rRNA database project was employed to classify each phylotype taxonomically, ensuring a sequence similarity exceeding 97%.

## 3. Results and Discussion

### 3.1. Batch Fermentation H_2_ Production from Xylose in the Presence of NaAc at Different Levels

**Figure 1:**
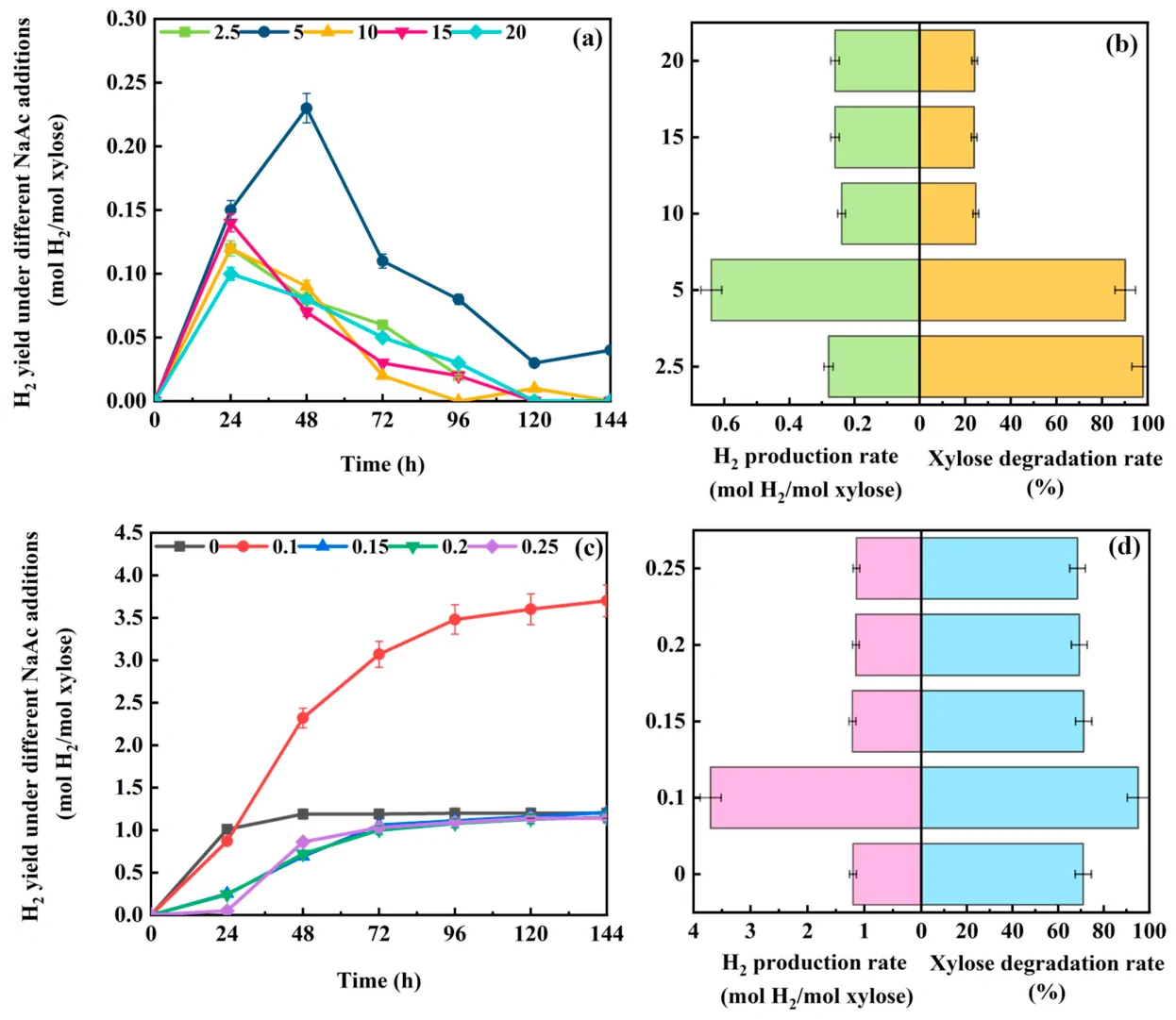
Effects of substrate and added NaAc concentration on DFHP from xylose. (**a**) Time curves of H_2_ production yield under different xylose concentrations; (**b**) total H_2_ production yield and xylose removal rate under different xylose concentrations; (**c**) time curves of H_2_ production yield under different NaAc additions at 10 g/L xylose; (**d**) total H_2_ production yield and xylose removal rate under different NaAc additions at 10 g/L xylose.

Different concentrations of NaAc and the substrate (xylose) were used to test the regulatory ability of the buffer solution system in terms of xylose H_2_ fermentation and determine the approximate concentration of NaAc required for subsequent experiments. As shown in Figure 1a,b, as the substrate concentration increased from 2.5 g/L to 5 g/L, H_2_ production showed an increasing trend. The maximum gas production was obtained at a substrate concentration of 5 g/L, which was 0.15 mol H_2_/mol xylose at 24 h, and production gradually stabilized at 0.64 mol H_2_/mol xylose after 48 h. With the further increase in substrate concentration to 10 g/L and 20 g/L, H_2_ production efficiency rapidly decreased, which was only 0.26 mol H_2_/mol xylose at 20 g/L, a decrease of 59.38%. This result is consistent with numerous previous reports; it is difficult for the biohydrogen production process enabled by dark fermentation to exceed a 10 g/L substrate concentration without any regulation, which indicates that hydrogen-producing microorganisms are sensitive to changes in the fermentation environment, such as over-acidification [27,28,29]. Notably, 10 g/L xylose exhibited a significantly lower substrate conversion efficiency compared to 5 g/L xylose, making it a suitable candidate for evaluating the regulatory effect of the buffer system under conditions with greater substrate loading. As shown in Figure 1c,d, the H_2_ fermentation broth was greatly affected by the addition of NaAc; as H_2_ production progressed, the regulated group’s gas production increased rapidly, reaching 2.32 mol H_2_/mol xylose with 0.1 mol NaAc added after 48 h, while the control group was only at 1.19 mol H_2_/mol xylose. At the end, the H_2_ production of the 0.1 mol/L NaAc-regulated group reached 3.7 mol H_2_/mol xylose, an increase of 2.08 times compared to the control group, suggesting that NaAc enhanced H_2_ production via pH stabilization or metabolic shifts. However, increasing the concentration of NaAc did not result in higher H_2_ production efficiency, as it remained even lower than that of the control group. It is speculated that the reason for this result is that the addition of NaAc has a buffering effect on the pH and achieves the best buffering effect at a concentration of 0.1 mol/L. As the concentration continues to increase, the pH continues to rise, and the buffering effect decreases, resulting in a decrease in H_2_ production. Based on this result, the substrate concentration for the subsequent semi-continuous-flow experiment was 10 g/L, and the NaAc concentration was 0.1 mol/L.

### 3.2. Performance of Semi-Continuous Two-Stage Biohythane Fermentation Process from Xylose with NaAc Buffer Regulation Under Different Conditions

#### 3.2.1. H_2_ and CH_4_ Production Performance During Different Fermentation Stages

**Figure 2:**
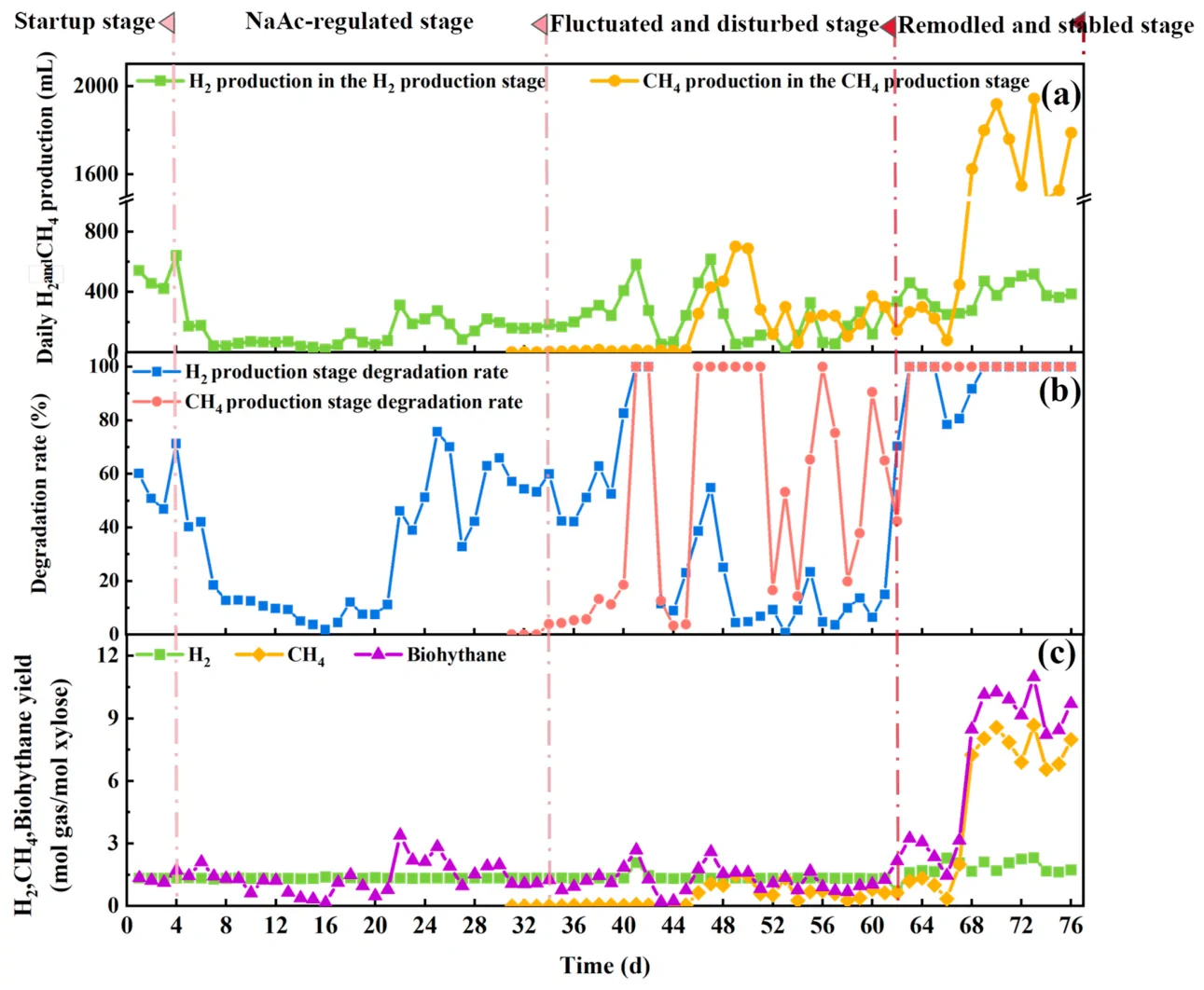
Performance of NaAc buffer solution regulation concerning biohythane production: (**a**) daily H_2_ and CH_4_ production at different fermentation stages; (**b**) substrate removal variations in H_2_ and CH_4_ fermentation stages; (**c**) daily H_2_ and CH_4_ and biohythane production yield observed during operational period.

During the 76-day continuous fermentation process, daily H_2_ and CH_4_ production demonstrated distinct phasic and coupled evolutionary patterns (Figure 2a). H_2_ production stabilized rapidly during the initial startup phase (days 1–4), reaching approximately 400 mL/d. Following the transition to semi-continuous feeding, H_2_ yield became variable. Notably, the supplementation of NaAc on day 22 restored H_2_ production, which stabilized at 397 ± 28 mL/d during the steady-state period (days 28–34, defined as daily production fluctuations within ±10% of the mean for at least five consecutive days), confirming the critical role of the buffering system in mitigating acid inhibition and sustaining hydrogenogenic activity [30]. However, progressively increasing the OLR challenged system stability. When xylose loading was raised to 1.5 g/d (days 35–42), daily H_2_ production exhibited initial inhibition followed by a sharp increase, peaking at 992 mL/d, with an average of 625 ± 187 mL/d over this period. A further increase to 2.25 g/d (days 43–45) induced severe inhibition, reducing H_2_ yield below 200 mL/d. Although shortening the HRT temporarily enhanced production to 1016 mL/d (days 46–47), the system ultimately destabilized under higher loads, with H_2_ production fluctuating widely between 35 and 705 mL/d from days 50 to 61, indicating limited resilience to organic overloading. This aligns with previous findings that excessive substrate concentrations can directly inhibit hydrogenase activity and reduce H_2_ production efficiency, with daily H_2_ production averaging 732 ± 94 mL/d during the final steady-state period (days 68–76) [31].

In contrast to the high sensitivity of H_2_ production, CH_4_ production displayed significant lag phases and greater systemic robustness. Following its initiation on day 30, CH_4_ production increased steadily after a brief lag, reaching 2000 mL/d by day 47, indicating the functional establishment of the methanogenic community. This startup lag is consistent with the typical 30–60 days required for the maturation and colonization of anaerobic methanogenic consortia. During the subsequent periods of high instability in the hydrogenogenic stage (days 48–67), daily CH_4_ production was affected (300–2030 mL/d) but demonstrated considerable metabolic buffering capacity. Once H_2_ production stabilized, CH_4_ yield rapidly rebounded and reached 4724 ± 361 mL/d during the final steady-state period (days 71–76), indicating that the methanogenic community maintained stable activity and metabolic resilience despite fluctuations in the upstream H_2_ production stage [32].

Gas composition analysis provided metabolic insights into these production trends (Figure S1a,b). During the stable operational phase with an OLR of 1.5 g/d (days 35–42), H_2_ content averaged 51.3 ± 6.3%, ranging from 40% to 59.47%, indicating stable hydrogenogenic activity. However, a slight decline in H_2_ content under excessive loading suggested the onset of substrate inhibition, consistent with reports that hydrogenase activity in hydrogen-producing bacteria can decrease by 15–45% when glucose concentrations exceed 2.5 g/L [31,33]. The CH_4_ content profile clearly reflected microbial community succession. The initial phase (<20%) corresponded to an immature methanogenic community, whereas a significant increase after day 47, culminating in stabilization at 55.1 ± 6.89% (peak 63.39%), marked the establishment of methanogenesis as the dominant pathway. Notably, the gaseous concentrations of H_2_ and CH_4_ remained relatively stable even when total gas production fluctuated, suggesting that their synthetic pathways remained intact, though overall metabolic flux was modulated by environmental conditions.

Overall, under the regulation of the sodium acetate buffer system, the two-stage biohythane process exhibited a clear “hydrogen-sensitive, methane-stable” metabolic division. The front-end hydrogenogenic stage responded sensitively and was susceptible to inhibition under load variations, whereas the terminal methanogenic stage demonstrated strong metabolic resilience and stable productivity. This dynamic coupling highlights the critical regulatory role of the buffering system in alleviating acid inhibition and facilitating efficient metabolic flux transfer between the two stages, providing a key basis for process optimization.

#### 3.2.2. Substrate Removal Efficiency

As shown in Figure 2b, during the initial batch operation (days 1–4), the H_2_-producing stage demonstrated an average xylose degradation efficiency of 57.33%, reaching a maximum of 71.33% on day 4, indicating effective bio-H_2_ conversion activity. The transition to semi-continuous feeding on day 5 resulted in a progressive decline in degradation performance, with efficiency decreasing to 1.88% by day 16, likely due to operational mode transition and microbial community adaptation. Furthermore, the degree of feeding is relatively low during this period, leading to the accumulation of undegradable xylose and volatile fatty acids, resulting in process inhibition [34]. The implementation of NaAc supplementation on day 22 significantly improved system performance, with degradation efficiency recovering to 75.78%. Subsequent operation showed fluctuating but generally improving degradation rates, achieving 100% substrate removal on day 42. However, this optimal performance was transient, as the OLR increasing from day 43 caused a dramatic reduction in degradation efficiency to 8.95%, suggesting that a sharp increase in the OLR shifted the fermentation pathway from a hydrogen-producing acetic/butyrate type to a solvent-producing (e.g., lactate/propionate) type, resulting in severe VFA accumulation that completely inhibited hydrogen-producing bacteria. The following 17-day period (days 45–61) maintained low degradation efficiencies (average 14.96%), also demonstrating the system’s sensitivity to loading shocks. Process optimization through HRT reduction, OLR adjustment, and continued NaAc supplementation effectively restored system performance, achieving nearly 100% degradation by day 63. Although minor fluctuations were observed between days 65 and 67 (average 83.64%), the overall performance demonstrated stable operation under the modified conditions.

The two-stage system configuration demonstrated complementary functionality, with the CH_4_-producing area effectively processing residual substrates from the H_2_-producing stage. During initial CH_4_-producing stage operation (days 31–40), xylose degradation efficiencies remained above 6.23% despite limited CH_4_ production, confirming ongoing hydrolytic and acidogenic activity. The complete degradation of residual xylose was achieved by day 41 and maintained during days 46–51, indicating system resilience. Significant variations in the OLR (days 52–62) affected methanogenic performance, with xylose degradation efficiencies fluctuating between 16.55% and 90.57%. System stability was re-established after day 64, with the consistent complete degradation of residual xylose, demonstrating the long-term operational robustness of the two-stage configuration.

#### 3.2.3. Specific H_2_ and CH_4_ Production

The variation in specific H_2_ and CH_4_ production yield per mol xylose in culture is shown in Figure 2c and Figure S1c. For the given substrate, H_2_ production performance remained stable with an average H_2_ yield of 1.26 mol/mol during the initial phase of system operation, consistent with the values reported in the literature [35,36]. However, during the unregulated phase (days 5–21), performance became highly variable (0.33–2.11 mol/mol), averaging 1.01 mol/mol. Significantly, the supplementation of NaAc (days 22–41) markedly enhanced process stability, sustaining H_2_ yields above 1.0 mol/mol and achieving peak values exceeding 3.0 mol/mol (75% of the theoretical maximum), with a 14.85% increase in average yield (1.16 mol/mol) compared to the unregulated phase. These results represent a substantial improvement over previous studies on H_2_ production from xylose through dark fermentation, including batch, semi-continuous, and continuous systems, and set a new benchmark for achievable yield under similar operational conditions [23,37,38]. This confirms the effectiveness of NaAc supplementation in mitigating acidogenic inhibition under moderate organic loading. However, the sharp decline to 0.17 mol/mol upon increasing substrate load (2.25 g/d, day 42) underscores the sensitivity of fermentative hydrogen production to acidogenic inhibition at high OLRs. The limited recovery (average 0.5 mol/mol) suggests that microbial consortia failed to fully acclimate to the elevated OLR, likely due to irreversible metabolic shifts toward solventogenesis or methanogenesis. Sasidhar et al. (2022) demonstrated that the OLR and HRT are interdependent parameters in anaerobic digestion, where the determination of one relies on the definition of the other, allowing for the adjustment of either through fine-tuning its counterpart as needed [39]. Consequently, subsequent operational adjustments (reduced HRT and OLR from day 62) not only restored H_2_ production but enhanced it, stabilizing at 2.0 mol/mol, a 72.41% improvement over the high-OLR phase and a 2-fold increase over the initial unregulated phase, demonstrating the reversibility of metabolic inhibition under optimized conditions. Notably, the modified strategy also triggered a dramatic shift in biohythane production, with yields surging from an average of 1.21 mol/mol (days 32–61) to a peak of 10.99 mol/mol (day 74) before stabilizing at 9.49 mol/mol with 1.91 mol H_2_/mol xylose and 7.63 mol CH_4_/mol xylose, suggesting that dynamic OLR control can synergistically maximize energy recovery by promoting syntrophic interactions between hydrogenogens and methanogens.

According to ECE calculations (Supplementary Table S4), the two-stage system achieves an overall ECE of 74.44%, which represents a 6.27-fold increase over single-stage H_2_ production (10.22%) and a 15.95% increase over single-stage CH_4_ production (64.2%). This performance can be primarily attributed to the NaAc buffering strategy, which effectively mitigated acid inhibition in the hydrogenogenic stage and facilitated efficient electron transfer between stages. Notably, the system maintained substantial H_2_ production alongside high CH_4_ yield, a distinct advantage over configurations where H_2_ production is significantly suppressed due to metabolic competition, as observed in some integrated systems [40]. When compared to single-stage processes, the efficiency gains are particularly evident. For instance, Chen et al. (2020) reported average ECE values of approximately 1.5% for hydrogenogenic systems and 9.8% for methanogenic systems, highlighting the synergistic effect achieved through stage coupling in the present study [41].

### 3.3. Response of pH and VFAs to Sodium Acetate Regulation in Two-Stage Biohythane Fermentation Process

**Figure 3:**
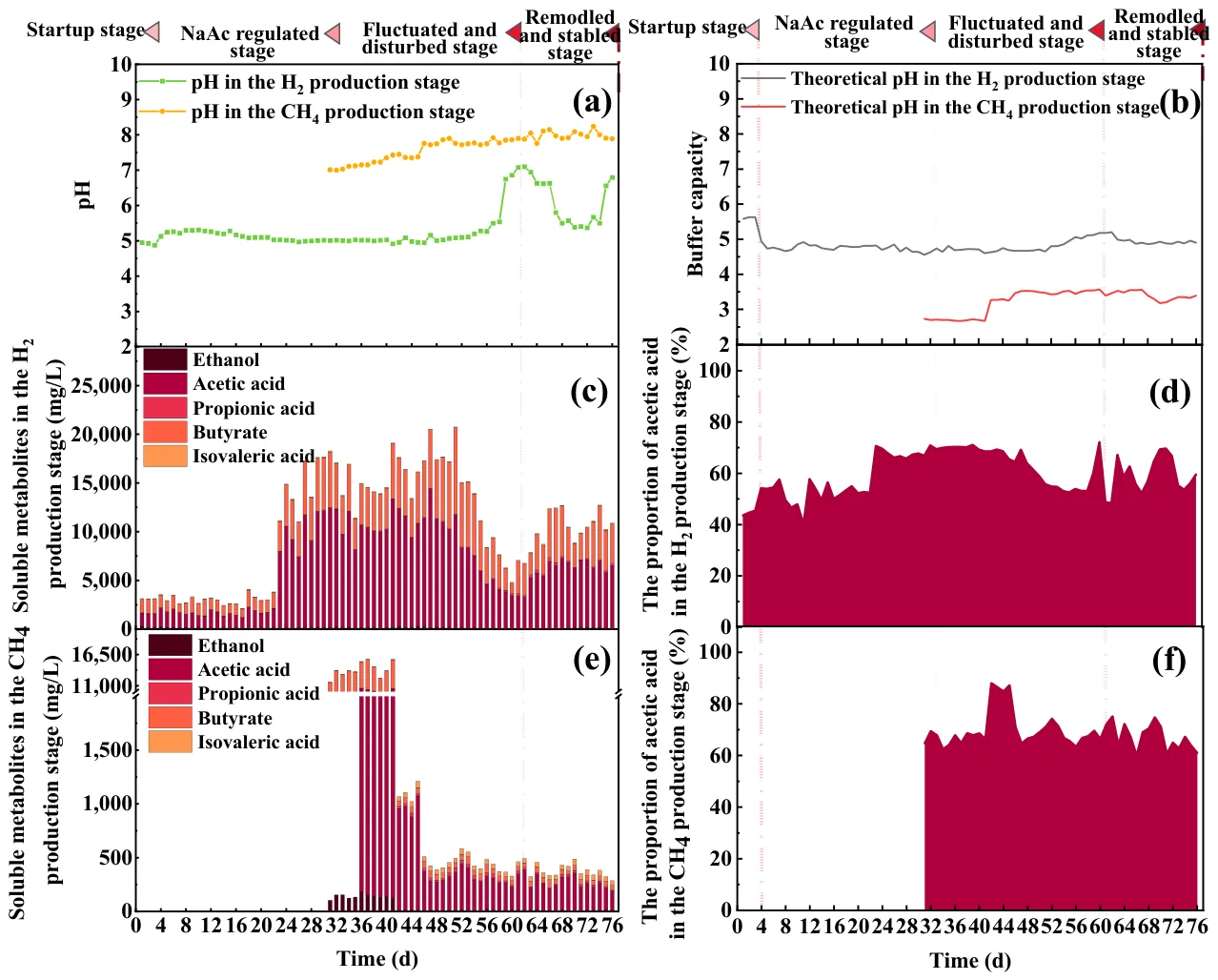
Time course of operational performance: (**a**) actual measured pH, (**b**) theoretical pH, (**c**) soluble metabolites in H_2_ production stage; (**d**) proportion of acetic acid in H_2_ production stage; (**e**) soluble metabolites in CH_4_ production stage; (**f**) proportion of acetic acid in CH_4_ production stage.

Monitoring and analyzing the dynamics of pH and volatile fatty acids (VFAs) are essential for precise system control and process stability prediction. These parameters directly regulate microbial metabolic activities and pathway distributions, thereby influencing overall system efficiency. As shown in Figure 3a,b, during system startup, the monitored pH was lower than the theoretical value, primarily attributable to the NaOH-mediated pH adjustment of the initial medium and partial neutralization of organic acids by phosphate-based alkaline additives. The monitored pH remained consistently higher than the values calculated based on VFA production from the fourth day onward. This indicates that, similarly to the batch hydrogen-producing system using microcrystalline cellulose described in Section 3.1, a dynamic acid–base equilibrium centered on the HAc/NaAc buffer was also established in this semi-continuous two-stage anaerobic fermentation process.

During the hydrogen-producing stage, the system stabilized at a pH of 6.23 after 60 days of operation, which was more than 2.81 units higher than the theoretical pH (<3.41), demonstrating that the buffer system not only counteracted the pH decline caused by organic acid accumulation but, more importantly, maintained the environment within the optimal activity range for hydrogen-producing microorganisms. This observation aligns with previous findings: the HAc/NaAc system establishes a dynamic equilibrium through reversible dissociation creating a favorable microenvironment that exceeds theoretical predictions by balancing proton release [18]. Metabolite analysis further confirmed that acetate and butyrate were the dominant liquid-phase end products during this stage (Figure 3c,d). Notably, the peak acetate concentration coincided with the period of the maximum daily hydrogen production, confirming that acetate-type fermentation was the dominant metabolic pathway driving efficient hydrogen generation. The addition of NaAc likely redirected metabolic flux, promoting both acetogenic and butyrogenic pathways, thereby collectively contributing to the generation of reducing equivalents and enhancing hydrogen conversion efficiency. The stable pH environment, coupled with an optimized metabolite profile, provided the necessary physicochemical and biochemical foundation for high-efficiency hydrogen production.

In the methanogenic stage, the system pH stabilized around 8.0, which is conducive to methanogen activity. Correspondingly, VFA degradation efficiency improved significantly after an initial low-activity period, eventually achieving the near-complete removal of acetate, butyrate, and ethanol (Figure 3e,f). However, methane production did not increase proportionally with VFA degradation during the initial phase, suggesting metabolic diversion where degraded substrates were preferentially used for microbial growth or converted into other intermediates. After 67 days of operation, the system entered a highly efficient and stable phase, characterized by efficient VFA conversion (≥97%) and increased methane production. These results demonstrate the dual regulatory function of the HAc/Ac^-^ buffer system in two-stage anaerobic fermentation. On the one hand, it dynamically maintains the optimal pH environment for each functional microbial community. On the other hand, it supplements the extracellular acetate pool and participates in the HAc/Ac^-^ equilibrium, potentially influencing VFA interconversion and methanogenic pathways. However, due to the chemical similarity between exogenous acetate from NaAc and endogenous acetate produced through xylose fermentation, it was impossible to quantitatively distinguish their individual impacts in this research. The supplemented NaAc should therefore be regarded primarily as a buffering agent and acetate pool regulator, rather than as a continuous external carbon source. Notably, the efficient removal of acetate in the methanogenic stage was not accompanied by a proportional increase in methane production, suggesting that acetate was channeled toward other metabolic pathways rather than acetoclastic methanogenesis. This hypothesis will be further examined in Section 3.4 in light of the microbial community composition and acetate mass balance.

### 3.4. The Shift in the Microbial Community Under NaAc Conditions

#### 3.4.1. Bacterial Community

**Figure 4:**
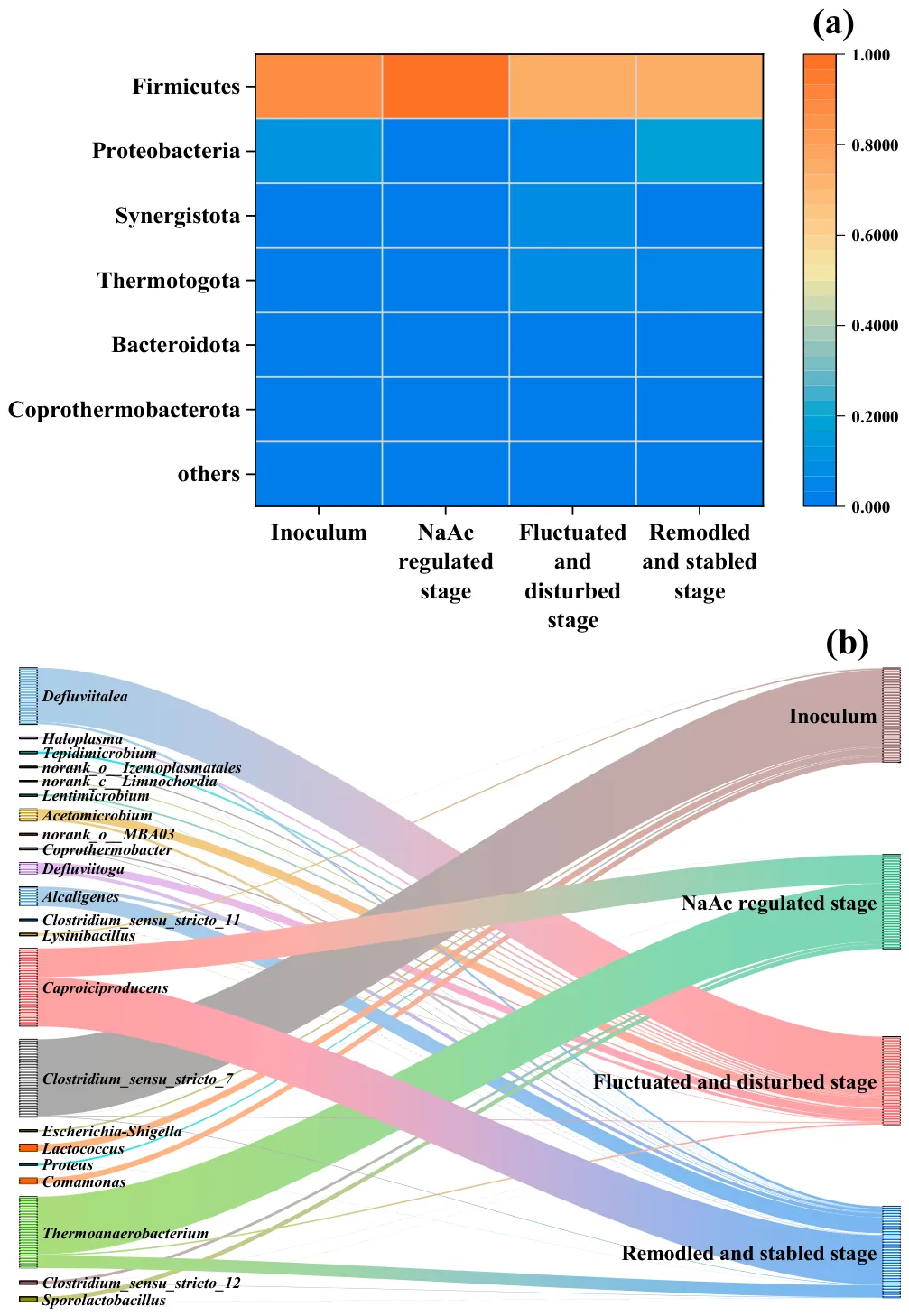
Distribution of bacterial community at phylum (**a**) and genus levels (**b**) under normal operation, methanogen-contaminated, and restored production conditions with NaAc regulation.

The degree of anaerobicity was 99.85% during normal operation, representing a 10.05% increase compared to the inoculum (Figure 4a). Bacterial phyla are commonly dominant in anaerobic digestion systems using lignocellulosic biomass, primarily responsible for hydrolysis and acidification [42]. Under NaAc regulation, subdominant phyla such as Proteobacteria, Synergistota, and Bacteroidota were sustained, suggesting that acetate amendment may enhance microbial diversity while maintaining functional specialization [43]. Following methanogenic backflow on day 53, the dominant Firmicutes decreased by 26.02%, while previously undetected phyla, including Synergistota (9.8%), Thermotogata (8.13%), Bacteroidota (2.24%), and Coprothermobacterota (1.54%), became significantly enriched. This shift likely resulted from elevated redox potential inhibiting strict anaerobes within Firmicutes and syntrophic acetate oxidation between Synergistota/Coprothermobacterota and methanogens [44]. After recovery, although Firmicutes increased slightly by 0.7%, Proteobacteria became the most responsive phylum, rising to 17.37%. In contrast, previously enriched phyla including Synergistota, Thermotogata, Bacteroidota, and Coprothermobacterota exhibited pronounced declines, indicating ecological niche reconstruction post-disturbance.

At the genus level (Figure 4b), *Thermoanaerobacterium* overwhelmingly dominated the inoculum (99.94%). During normal operation, a distinct shift occurred, with *Thermoanaerobacterium* and *Caproiciproducens* emerging as the dominant genera (60.87% and 30.16%, respectively), reflecting a community adaptation to xylose fermentation. *Caproiciproducens* is recognized for its ability to utilize xylose for hexanoic acid production [45], while *Thermoanaerobacterium* contributes to hydrogen generation from carbohydrates [46]. Following the methanogenic backflow event, the abundance of *Caproiciproducens* decreased precipitously from 30.12% to 0.17%. This drastic decline is likely attributable to pH fluctuations falling outside the optimal range (pH 6.0–7.0), a finding consistent with the pH variations detailed in Section 3.3 and confirming environmental pH as a critical regulator for this functional genus. Conversely, *Defluviitalea* became dominant (56.91%), a genus known for ethanol production without concomitant hydrogen generation, which aligns with the observed collapse in hydrogen yield. Concurrently, *Acetomicrobium* and *Defluviitoga* increased from undetectable levels to approximately 10%, likely benefiting from liberated ecological niches and their inherent metabolic versatility [47]. Upon restoration with sodium acetate (NaAc), the community structure shifted again: *Defluviitalea* decreased sharply to 2.61%, while *Caproiciproducens* re-established dominance (52.08%), indicating a metabolic reorientation toward acetate-based pathways. This period also saw a marked enrichment in *Alcaligenes* (16.94%), a genus associated with acetate oxidation [48,49]. *Thermoanaerobacterium* recovered only partially (12.86%), and *Acetomicrobium* and *Defluviitoga* declined dramatically. These collective changes demonstrate NaAc-mediated selection for acetate-utilizing taxa and underscore a system-wide metabolic transition toward efficient hydrogenogenic metabolism.

#### 3.4.2. Microbial Community Composition in Methane-Producing Region

**Figure 5:**
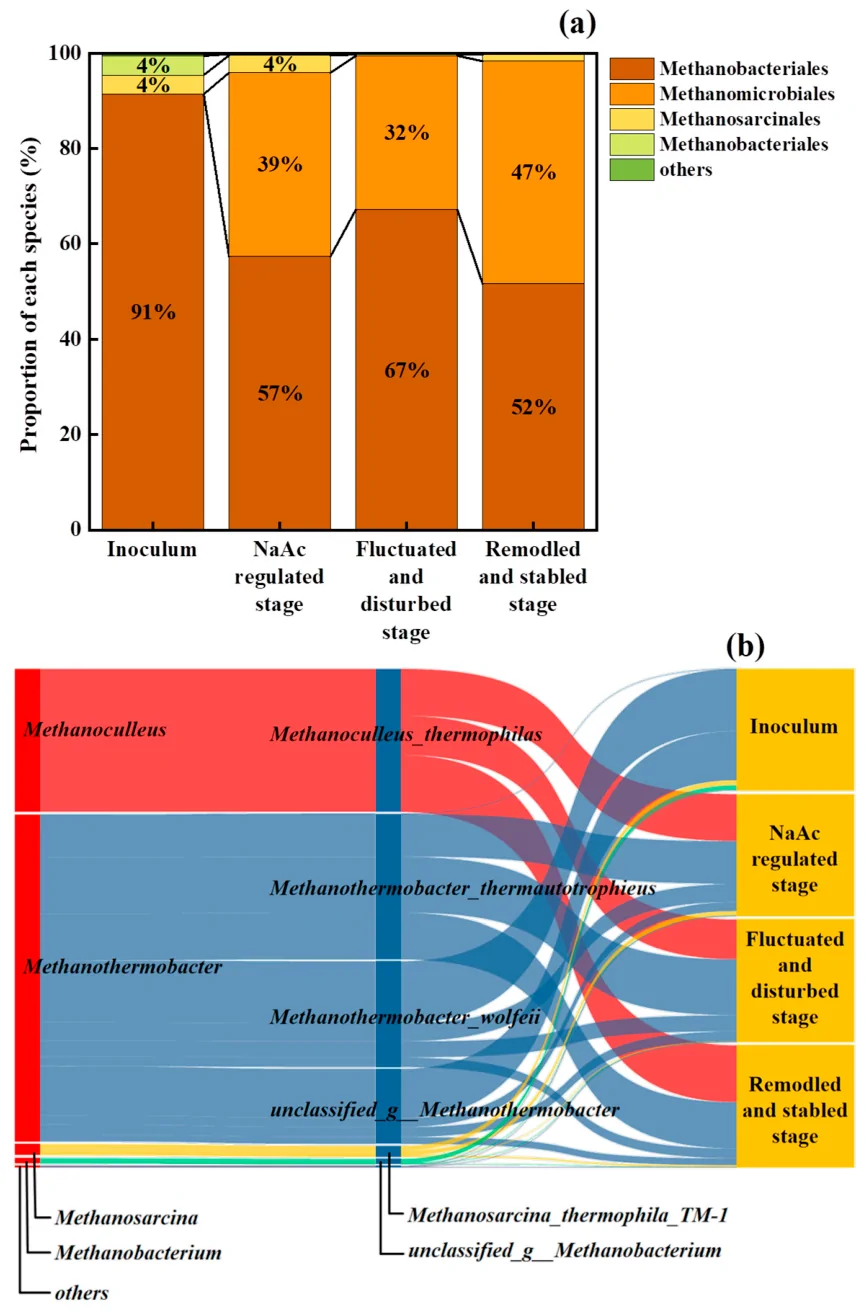
Distribution of archaeal community at phylum (**a**) and genus levels (**b**) under normal operation, methanogen-contaminated, and restored production conditions with NaAc regulation.

Archaea, as key methanogenic microorganisms, critically regulate CH_4_ production efficiency through their compositional dynamics and metabolic activities during anaerobic fermentation [43]. As shown in Figure 5a,b, the inoculum was overwhelmingly dominated by hydrogenotrophic *Methanobacteriales* (91.37%), with minor *Methanosarcinales* (4.03%). Following system startup, the community structure underwent significant restructuring. The relative abundance of *Methanobacteriales* sharply declined to 57.38%, while previously undetectable *Methanomicrobiales* emerged as a significant component (38.52%), suggesting that they exhibited superior adaptability to the prevailing conditions (e.g., H_2_/CO_2_ availability) during active methanogenesis [50]. Notably, acetoclastic *Methanosarcinales* consistently diminished to <0.3% throughout the operation, indicating that the acetoclastic pathway contributed minimally to methanogenesis. At the genus level, the community shifted from initial dominance by *Methanothermobacter wolfeii* (50.87%) and unclassified *Methanothermobacter* (40.29%) to a stable coexistence of *Methanoculleus thermophilus* (46.66%) and *Methanothermobacter thermautotrophicus* (37.94%). Both are hydrogenotrophic methanogens, and their coexistence reflects cooperative H_2_/CO_2_ utilization rather than a shift toward acetoclastic methanogenesis. The persistent dominance of the hydrogenotrophic pathway, despite the acetate-rich effluent from the H_2_-producing stage, can be attributed to several mutually reinforcing factors. First, the inoculum was overwhelmingly dominated by hydrogenotrophic methanogens, which likely directed subsequent community assembly through priority effects [51,52]. Second, the slow-growing acetoclastic Methanosarcina was nearly completely washed out under continuous-flow conditions (<0.3%), preventing its re-establishment even when acetate became available. Third, acetate in the methanogenic stage was largely channeled toward chain elongation rather than acetoclastic methanogenesis. The specific enrichment in *Caproiciproducens* (30.16% of Firmicutes), a genus capable of converting acetate and ethanol to medium-chain fatty acids such as caproate [45,53], suggests that acetate was largely channeled toward chain elongation rather than acetoclastic methanogenesis. This is further confirmed by the semi-quantitative acetate mass balance (Supplementary Tables S2 and S3): of the total acetate pool, only 5.2 mmol was consumed via acetoclastic methanogenesis (<0.3% of total CH_4_), whereas approximately 1015 mmol was consumed via chain elongation, and 187.5 mmol was assimilated into biomass. The mass balance closure was 65.6%, with the unaccounted fraction likely retained as Ac^-^ or converted to unidentified intermediates. Fourth, the residual xylose carried over by backflow sustained endogenous H_2_/CO_2_ production through continued fermentation by non-methanogenic populations, thermodynamically favoring the hydrogenotrophic pathway [54]. Collectively, the competitive consumption of acetate by chain elongation and the sustained endogenous H_2_ supply for the hydrogenotrophic pathway formed a self-reinforcing loop that preserved the dominance of hydrogenotrophic methanogens throughout the operation. These hypotheses warrant further investigation through a targeted analysis of chain elongation metabolites in future studies.

In summary, based on the analysis of reactor operating parameters and comparative experimental data, the addition of an acetate/acetic acid buffer solution was demonstrated to significantly enhance biohythane production efficiency. The underlying mechanisms are proposed as follows: (1) The buffering system effectively maintained the reactor pH within an optimal range of 6.5–7.2, providing favorable growth conditions for hydrogen-producing microorganisms. (2) Enhanced cellular tolerance may be attributed to the penetration of protonated acetic acid molecules through cell membranes, potentially reinforcing the proton motive force and improving acid resistance. (3) Under high acetate concentration stress, microbial community optimization occurred, with *Caproiciproducens* dominating the H_2_ production zone, accompanied by *Alcaligenes* and *Thermoanaerobacterium*, while *Defluviitalea* showed the most pronounced shift during system disturbance; *Methanoculleus thermophilus* became the predominant archaeal species in the methanogenic zone, ultimately establishing a stable two-stage H_2_ and CH_4_ cogeneration system. These proposed mechanisms warrant further validation through targeted physiological, transcriptomic, or proteomic analyses in future studies.

## 4. Conclusions

This study demonstrates that sodium acetate (NaAc) regulation significantly enhances two-stage biohythane production from xylose. The in situ HAc/Ac^−^ buffer pair maintained the pH of the H_2_-producing stage at 6.23 and that of the CH_4_-producing stage at ~8.0, alleviating acid inhibition and enabling stable long-term operation. Under optimized conditions (HRT 1.5 d, OLR ~1.5 g/L), the maximum biohythane yield reached 10.99 mol/mol (≈20% H_2_, 80% CH_4_), with a stable yield of 9.49 mol/mol. The overall energy conversion efficiency (ECE) reached 74.44%, 7.27-fold higher than that of single-stage H_2_ production (10.24%) and 1.16-fold higher than that of single-stage CH_4_ production (64.20%). Microbial analysis revealed that NaAc drove directional community succession. In the H_2_-producing stage, *Caproiciproducens* (52.08%) dominated after recovery, accompanied by *Alcaligenes* (16.94%) and *Thermoanaerobacterium* (12.86%). In the CH_4_-producing stage, hydrogenotrophic methanogens prevailed, with *Methanoculleus thermophilus* at 46.66% and *Methanothermobacter thermautotrophicus* at 37.94%. This indicates that acetate was mainly channeled toward chain elongation rather than acetoclastic methanogenesis, and NaAc functioned primarily as a buffer and supplemental acetate pool rather than a direct carbon source. These findings clarify the process, metabolic, and microbial mechanisms by which NaAc regulates two-stage biohythane fermentation. The strategy offers a promising route for converting xylose-rich biomass and industrial waste streams into biohythane.

## Data Availability

The raw data supporting the conclusions of this article will be made available by the authors on request.

## Supplementary Materials

The following supporting information can be downloaded at https://www.mdpi.com/article/10.3390/microorganisms14102389/s1, Text S1. DF medium and enrichment methods; Table S1. Key operating conditions during continuous operation: Loading rate and feedstock concentration; Table S2. Semi-quantitative mass balance of supplemented sodium acetate (NaAc) in the two-stage biohythane fermentation process; Table S3. Semi-quantitative mass balance table; Table S4. Overall ECE of the entire experiment; Figure S1. Performance of NaAc buffer solution regulation concerning biohythane production: (a) daily biogas production at different fermentation stage; (b) the variations in H_2_ and CH_4_; (c) daily substrate load and the end content of xylose dosage.
