# Supplementary material for: In Situ Formation of HAc/Ac− Buffer Pair via Sodium Acetate Addition Enhances Two-Stage Continuous Biohythane Production from Xylose: Process Performance and Microbial Community Dynamics
Source: Microorganisms. 2026 Oct 19;14(10):2389. doi: 10.3390/microorganisms14102389 (PMC13707745; doi:10.3390/microorganisms14102389)
Supplement: Supplementary file 1 [file microorganisms-14-02389-s001.zip › microorganisms-4584444-supplementary.pdf]

**Supporting Information**

# In Situ Formation of HAc/Ac⁻ Buffer Pair via Sodium Acetate Addition Enhances Two-Stage Continuous Biohythane Production from Xylose: Process Performance and Microbial Community Dynamics

Xiaoyi Feng[1], Kaini Dong[1], Qiulan Xie[1], Lili Dong[1, 2*], Jiwen Wu[1], Guangli Cao[1*]

1 State Key Laboratory of Urban Water Resources and Environment, School of Environment, Harbin Institute of Technology, Harbin 150090, China

2 Key Laboratory of Agro-Forestry Environmental Processes and Ecological Regulation, School of Environmental Science and Engineering, Hainan University, Haikou, 570228, China

* Correspondence:
Dr. Lili Dong
School of Environmental Science and Engineering, Hainan University(HNU), 58 Renmin Road, Haikou, Hainan, 570228, China
E-mail: dongll0569@hainanu.edu.cn
Phone & Fax: 86- 98-66269468

**Summary: 8 pages, 1 texts, 1 figure, and 1 Video**

**Text S1. DF medium and enrichment methods**

Based on previous studies (Cao et al., 2014), the culture medium (MA) for dark fermentation hydrogen production includes: L-cysteine (0.5 g $L^{-1}$), $NH_4Cl$ (1 g $L^{-1}$), $K_2HPO_4$ (1 g $L^{-1}$), $KH_2PO_4$ (1 g $L^{-1}$), $MgCl_2$ (0.5 g $L^{-1}$), KCl (0.2 g $L^{-1}$), Yeast extract (2 g $L^{-1}$), Peptone (2 g $L^{-1}$), Accelerator (1 g $L^{-1}$). Prior to experimentation, the initial pH of the medium was adjusted to 7.0, and the medium underwent deoxygenation and sterilization [18].

**Supporting Table S1.** Key operating conditions during continuous operation: Loading rate and feedstock concentration.

| Operation time (d) | Influnt liquid volume (mL/d) | Hydraulic retention time (d) | xylose quantity (g/d) |
|---|---|---|---|
| 1-4 | 900 | 1 | 4.5 |
| 5, 6 | 100 | 9 | 1 |
| 7-9 | 200 | 4.5 | 0.2, 1 |
| 10-18 | 100 | 9 | 1 |
| 18-38 | 200 | 4.5 | 1-1.5 |
| 39-59 | 150-300 | 3-6 | 1.5-3.15 |
| 60, 61 | 200 | 4.5 | 1.5 |
| 62-65 | 600 | 1.5 | 1.5 |
| 66-69 | 900 | 1 | 1.5 |
| 70-76 | 600 | 1.5 | 1.5 |

**Video S1.** Semi-quantitative mass balance of supplemented sodium acetate (NaAc) in the two-stage biohythane fermentation process

A semi-quantitative mass balance of the supplemented NaAc was established based on the measured VFA profiles, gas production, and biomass yield. Because exogenous acetate (derived from NaAc) and endogenous acetate (generated from xylose fermentation) have identical chemical structures, they cannot be distinguished by routine GC analysis. Therefore, the following calculation is semi-quantitative and is intended to clarify the primary role of NaAc in the two-stage system.

## 1. Basic parameters and unit conversions

**Supporting Table S2.** Semi-quantitative mass balance of supplemented sodium acetate (NaAc) in the two-stage biohythane fermentation process

| Parameter | Value | Unit |
|---|---|---|
| Total NaAc added | 164.8 | g |
| Molecular weight of NaAc | 82.03 | g/mol |
| Exogenous acetate input from NaAc | 164.8/82.03=2.009164.8/82.03=2.009 | mol |
| Exogenous acetate input | 2009 | mmol |
| Total feed volume in $H_2$-producing stage | 20.09 | L |
| Total effluent volume after $CH_4$-producing stage | 18.55 | L |
| Total xylose fed | 102.9 | g |
| Xylose molecular weight | 150.13 | g/mol |
| Total xylose fed | 102.9/150.13=0.685102.9/150.13=0.685 | mol |
| Total $H_2$ produced | 17.20 | L |
| Total $H_2$ produced | 17.20/22.4=0.76817.20/22.4=0.768 | mol |
| Total $CH_4$ produced | 38.62 | L |
| Total $CH_4$ produced | 38.62/22.4=1.72438.62/22.4=1.724 | mol |
| Protein concentration in $CH_4$-producing stage | 228.35 | mg/L |

## 2. Total acetate in the $H_2$ production stage

After NaAc supplementation, the average acetate concentration in the $H_2$ production stage was:

$$\overline{C}_{acetate,H_2} = 8427 mg/L$$

The total acetate input to the $H_2$ production stage was therefore:

$m_{acetate,H2}$=8427×20.09=169258 *mg*=169.26 *g*=2821 *mmol*

Among this total, exogenous acetate from NaAc accounted for 2009 mmol, and endogenous acetate generated from xylose fermentation was estimated as:

$n_{endogenous}$=2821−2009=812 *mmol*

Thus, exogenous acetate accounted for approximately 71.2% of the total acetate pool in the $H_2$ production stage, while endogenous acetate accounted for approximately 28.8%.

**3. Residual acetate in the effluent of the $CH_4$ production stage**

The average acetate concentration in the $CH_4$ production stage effluent was:

$$\overline{C}_{acetate,effluent} = 359.0 mg/L$$

The residual acetate in the effluent was:

$m_{acetate,effluent}$=359.0×18.55=6659 *mg*=6.66 *g*=111 *mmol*

**4. Acetate consumed via chain elongation**

The average butyrate concentration in the $H_2$ production stage was:

$$\overline{C}_{butyrate,H_2} = 4493.5 mg/L$$

The total butyrate input to the $CH_4$ production stage was:

$m_{butyrate,H2}$=4493.5×20.09=90274 *mg*=90.27 *g*=1026 *mmol*

The average butyrate concentration in the $CH_4$ production stage effluent was:

$$\overline{C}_{butyrate,effluent} = 51.9 mg/L$$

$m_{butyrate,effluent}$=51.9×18.55=963 *mg*=0.963 *g*=10.9 *mmol*

The net butyrate removal was therefore:

$n_{butyrate,removed}$=1026−10.9=1015.1 $mmol$

Based on the chain elongation stoichiometry:

butyrate+acetate→caproate

1 mol of butyrate consumption corresponds to 1 mol of acetate consumption. Therefore, the acetate consumed via chain elongation was estimated as:

$n_{acetate,chain\ enlongation}$ ≈1015 $mmol$

Because caproate was not quantified in this study, this value is semi-quantitative. Subsequent research will focus on the absolute quantification of carbon chain elongation.

**5. Acetate consumed via acetoclastic methanogenesis**

The total $CH_4$ produced was 1724 mmol. The methanogenic community was dominated by hydrogenotrophic methanogens, while acetoclastic *Methanosarcina* accounted for less than 0.3% of the archaeal community. Taking a conservative upper limit of 0.3%:

$f_{acetoclastic}$=0.003

$n_{CH4,acetoclastic}$=1724×0.003=5.2 $mmol$

This contribution is negligible.

**6. Acetate assimilated into biomass**

The protein concentration in the $CH_4$ production stage was 228.35 mg/L, and the effluent volume was 18.55 L:

$m_{protein}$=228.35×18.55=4236 $mg$=4.24 $g$

Assuming protein accounts for 50% of cell dry weight:

$m_{biomass}$=4.24/0.5=8.47 $g$

Using a typical microbial formula $C_5H_7O_2N$ (molecular weight 113 g/mol):

$n_{biomass}$=8.47/113=0.075 *mol*=75 *mmol*

Approximately 2.5 mol of acetate are required per mol of biomass synthesized:

$n_{acetate,biomass}$=75×2.5=187.5 *mmol*

This value includes both exogenous and endogenous acetate and cannot be further differentiated.

**Supporting Table S3.** Semi-quantitative mass balance table

| Item | Formula / basis | Value |
|---|---|---|
| **Input** | | |
| NaAc added | 164.8 | 164.8 |
| Exogenous acetate input | 164.8 / 82.03 | 2009 |
| Total acetate in $H_2$-producing stage | 8427 mg/L × 20.09 L | 2821 |
| Endogenous acetate (estimated) | 2821 − 2009 | 812 |
| **Output / sinks** | | |
| Residual acetate in effluent | 359.0 mg/L × 18.55 L | 111 |
| Acetoclastic methanogenesis | 1724 mmol $CH_4$ × 0.3% | 5.2 |
| Chain elongation | Net butyrate removal 1015 mmol × 1 | 1015 |
| Biomass assimilation | 75 mmol biomass × 2.5 | 187.5 |
| Total identified sinks | 111 + 5.2 + 1015 + 187.5 | 1318.7 |
| Unaccounted exogenous acetate | 2009 − 1318.7 | 690.3 |
| **Mass balance closure** | 1318.7 / 2009 | 65.6 |

The exogenous acetate input from NaAc was 2009 mmol, accounting for approximately 71.2% of the total acetate pool in the $H_2$ production stage, while endogenous acetate generated from xylose fermentation was estimated at 812 mmol, accounting for approximately 28.8%. The identified acetate sinks included residual acetate in the effluent (111 mmol), acetoclastic methanogenesis (5.2 mmol, <0.3% of total $CH_4$), chain elongation (1015 mmol, estimated from net butyrate removal), and biomass assimilation (187.5 mmol). The mass balance closure was 65.6%, with the unaccounted fraction likely retained in the reactor as $Ac^-$ to maintain the $HAc/Ac^-$

buffer equilibrium or converted to unidentified intermediates such as caproate or butanol.

Because acetate is a central metabolic intermediate and exogenous and endogenous acetate cannot be distinguished by routine GC analysis, this mass balance is semi-quantitative. Nevertheless, the results support that the primary role of NaAc is buffering rather than serving as a major external carbon source. Future studies using $^{13}C$-labeled acetate or metabolic flux analysis are needed for precise quantification.

**Supporting Table S4. Overall ECE of the entire experiment**

The overall energy conversion efficiency (ECE) was recalculated by clearly specifying that the energy inputs include both xylose and NaAc, and the energy outputs include both $H_2$ and $CH_4$. The calculation was performed using the total gas production and total substrate input over the entire experimental period.

**Supporting Table S4.** Overall ECE of the entire experiment.

| Parameter | Value | Unit |
|---|---|---|
| Total $H_2$ production | 17200.85 | mL |
| Total $CH_4$ production | 38622.49 | mL |
| Total xylose fed | 102.9 | g |
| Total NaAc added | 164.8 | g |
| Heating value of $H_2$ ($Q_{H2}$) | 12.86 | J/mL |
| Heating value of $CH_4$ ($Q_{CH4}$) | 35.9 | J/mL |
| Heating value of xylose ($Q_{xylose}$) | 4000 | J/g |
| Energy equivalent of NaAc ($Q_{NaAc}$) | 10608 | J/g |
| ECE of overall two-stage process ($H_2$ + $CH_4$) | 1607.7/2159.8×100%=74.44 | % |
| ECE of $H_2$ production stage alone | 221.2/2159.8×100%=10.24 | % |
| ECE of $CH_4$ production stage alone | 1386.5/2159.8×100%=64.20 | % |

## Supporting Figure

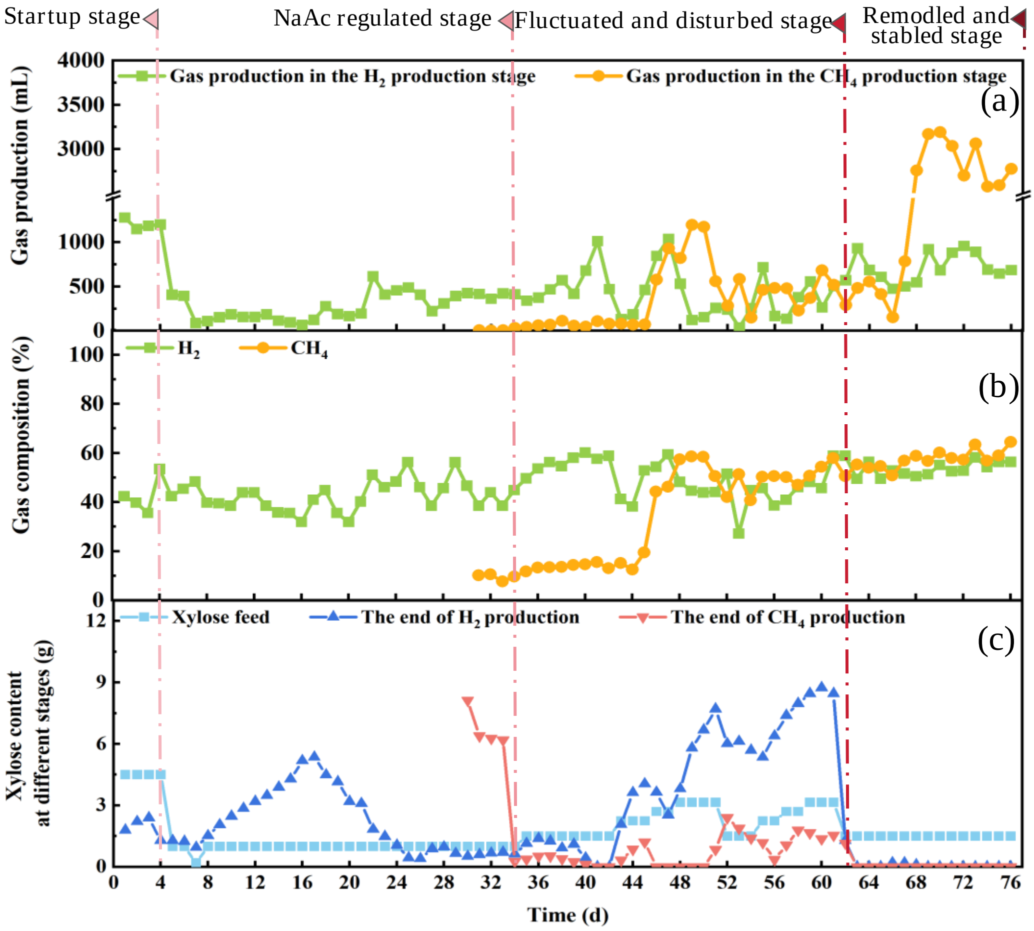


**Figure S1.** Performance of NaAc buffer solution regulation concerning biohythane production: (a) daily biogas production at different fermentation stage; (b) the variations in $H_2$ and $CH_4$; (c) daily substrate load and the end content of xylose dosage.
